# Anti-CCL2 therapy reduces oxygen toxicity to the immature lung

**DOI:** 10.1038/s41420-024-02073-5

**Published:** 2024-07-03

**Authors:** Tayyab Shahzad, Ying Dong, Nina K. Behnke, Julia Brandner, Anne Hilgendorff, Cho-Ming Chao, Judith Behnke, Saverio Bellusci, Harald Ehrhardt

**Affiliations:** 1 Department of General Pediatrics and Neonatology, Justus-Liebig-University and Universities of Giessen and Marburg Lung Center (UGMLC), German Lung Research Center (DZL), Feulgenstrasse 12, Giessen, Germany; 2 Division of Neonatology, University Children’s Hospital, Perinatal Center, Ludwig-Maximilians-University, Campus Großhadern, Marchioninistrasse 15, Munich, Germany; 3 Institute for Lung Health and Immunity and Comprehensive Pneumology Center, Helmholtz Zentrum München, German Center for Lung Research (DZL), Munich, Germany; 4 Department of Pediatrics, Helios University Medical Center, Witten/Herdecke University, Heusnerstrasse 40, 42283 Wuppertal, Germany; 5 Department of Internal Medicine II, Universities of Giessen and Marburg Lung Center (UGMLC), Cardio-Pulmonary Institute (CPI), Germany German Lung Research Center (DZL), Aulweg 130, Giessen, Germany; 6 Division of Neonatology and Pediatric Intensive Care Medicine, Department of Pediatrics and Adolescent Medicine, University Medical Center Ulm, Ulm, Germany

**Keywords:** Respiratory tract diseases, Chronic inflammation, Paediatric research

## Abstract

Oxygen toxicity constitutes a key contributor to bronchopulmonary dysplasia (BPD). Critical step in the pathogenesis of BPD is the inflammatory response in the immature lung with the release of pro-inflammatory cytokines and the influx of innate immune cells. Identification of efficient therapies to alleviate the inflammatory response remains an unmet research priority. First, we studied macrophage and neutrophil profiles in tracheal aspirates of *n* = 103 preterm infants <29 weeks´ gestation requiring mechanical ventilation. While no differences were present at birth, a higher fraction of macrophages, the predominance of the CD14^+^CD16^+^ subtype on day 5 of life was associated with moderate/severe BPD. Newborn CCL-2^−/−^ mice insufficient in pulmonary macrophage recruitment had a reduced influx of neutrophils, lower apoptosis induction in the pulmonary tissue and better-preserved lung morphometry with higher counts of type II cells, mesenchymal stem cells and vascular endothelial cells when exposed to hyperoxia for 7 days. To study the benefit of a targeted approach to prevent the pulmonary influx of macrophages, wildtype mice were repeatedly treated with CCL-2 blocking antibodies while exposed to hyperoxia for 7 days. Congruent with the results in CCL-2^−/−^ animals, the therapeutic intervention reduced the pulmonary inflammatory response, attenuated cell death in the lung tissue and better-preserved lung morphometry. Overall, our preclinical and clinical datasets document the predominant role of macrophage recruitment to the pathogenesis of BPD and establish the abrogation of CCL-2 function as novel approach to protect the immature lung from hyperoxic injury.

## Introduction

The pulmonary inflammation in the immature lung provoked by oxygen toxicity and reactive oxygen species formation as initial step constitutes the main cause of bronchopulmonary dysplasia (BPD) in the preterm infant [1, 2]. It is characterized by an innate immune response with the influx of mainly macrophages and neutrophils and the release of pro-inflammatory cytokines that has been detailed mechanistically in preclinical models of hyperoxia exposure [3, 4]. The extent of lung injury and the distortion of further lung development determine the severity of BPD that results in life-long restrictions in lung function as lung catch-up growth is not feasible [5–7].

Based on observational studies in preterm infants and pathomechanistic studies in preclinical models, the approach of targeting the pulmonary inflammatory response has attracted particular attention during the recent years as has been successfully established in other pediatric and adult lung diseases [6, 8, 9]. Overall, targeting IL1β activation and subsequent formation of the inflammasome is the so far best studied targeted approach and results in preclinical rodent models univocally confirm reduced inflammation and better-preserved lung morphometry [10, 11]. This has been funded by tracheal aspirate studies where specific changes in the IL1 genes early after birth were detected in infants developing BPD [12]. In contrast, studies with TNF-α and IL-6 knockout mice led to more severe lung injury or contradictory results respectively [13–15]. Respecting the overall results on this topic, it comes more and more clear that overstimulation of the pro-inflammatory signaling pathways leads to lung injury but simultaneously physiologic activation status of these pathways is a prerogative for physiologic lung growth [3]. This revised pathomechanistic concept delivers the foundation why suppression of single pathways results in disbalance of the complex signaling network in the immature lung with more severe inflammation and lung injury affecting the alveolar, mesenchymal and vascular compartments [3, 13, 16]. This is why the number of available drugs with documented efficacy to prevent BPD is so far limited [17]. Newly, concerns arise that globally dampening the inflammatory response with corticosteroids results in more severe restrictions in lung function later in life despite the well-documented short-term efficacy to improve the gas exchange [18]. These clinical are substantiated by preclinical studies in the rodent model [19].

Observational studies in preterm infants during the last two decades prevailed that the presence of macrophages in tracheal aspirate samples of ventilated preterm infants was associated with BPD [3, 20, 21]. Measurements of CCL-2 as chemoattractant for lung macrophage recruitment prevailed congruent results with increased levels in tracheal aspirates in those infants developing BPD [22]. Preclinical studies in the newborn hyperoxia rodent model uniformly showed that macrophage recruitment to the immature lung is a critical step in the pathogenesis of BPD. Treatment with blocking antibodies against the CCR2 receptor or CCL2 protected from the excessive influx of macrophages into the lung and displayed reduced structural abnormalities following exposure to hyperoxia [23, 24]. But recently, concerns were raised in newborn myeloid depleted mice exposed to hyperoxia about the safety of such a targeted approach where the increased lung injury was attributed to decreased IFNγ and increased MMP9 [25, 26].

We recently provided further insights into monocyte subset dynamics in the peripheral blood of preterm infants <32 weeks´ gestation at birth and longitudinally until two weeks after the end of invasive mechanical ventilation (MV). Infants developing BPD prevailed a higher number of CD14^+^CD16^+^ non-classical monocytes at birth and during the longitudinal course of MV with supplemental oxygen that were identified as major pro-inflammatory source [27]. Thereby, specific changes in the cytokine and growth factor cytokine profile in the cord blood were associated with the predominance of the non-classical monocyte signature and CCL2 was identified as key upregulated cytokine in the statistical model. Lastly, moderate/severe BPD cases were characterized by the coincidence of predominantly pro-inflammatory pathway activation and increased reactive oxygen formation as initial hallmark of oxygen toxicity [27].

Now, we expanded these previous insights within a translational approach and focus on the characteristics of the lung cellular inflammatory response and the non-classical macrophages in preterm infants <29 weeks´ gestation exposed to MV with oxygen rich gas. We detailed the importance of macrophage recruitment to the immature lung for morphologic alterations in newborn CCL2^−/−^ mice exposed to different concentrations of oxygen. Finally, the therapeutic potential of blocking CCL2 function in vivo was tested in the newborn hyperoxia mouse model.

## Results

### Higher fraction of macrophages and predominance of a CD14^+^CD16^+^ subtype in tracheal aspirates of preterm infants developing moderate/severe BPD

We studied the effects of mechanical ventilation with oxygen rich gas on the pro-inflammatory innate immune response in tracheal aspirates of *n* = 114 preterm infants <29 weeks´ gestation that required intubation and mechanical ventilation after birth. Of the total cohort, 11 infants died before 36 weeks´ gestation where severity of BPD could not be assessed and *n* = 68 infants of the original cohort still depended on mechanical ventilation (MV) with oxygen rich gas (HOX) on day 5 of life. Patients had a median gestational age of 26 + 0 and a median birth weight of 724 grams. Preterm infants were provided with supplemental oxygen as recommended to keep their oxygen saturations within the target of 85–95% and the fraction of oxygen varied largely between the infants reflecting less or more severely compromised gas exchange [2, 28].

**Fig. 1:**
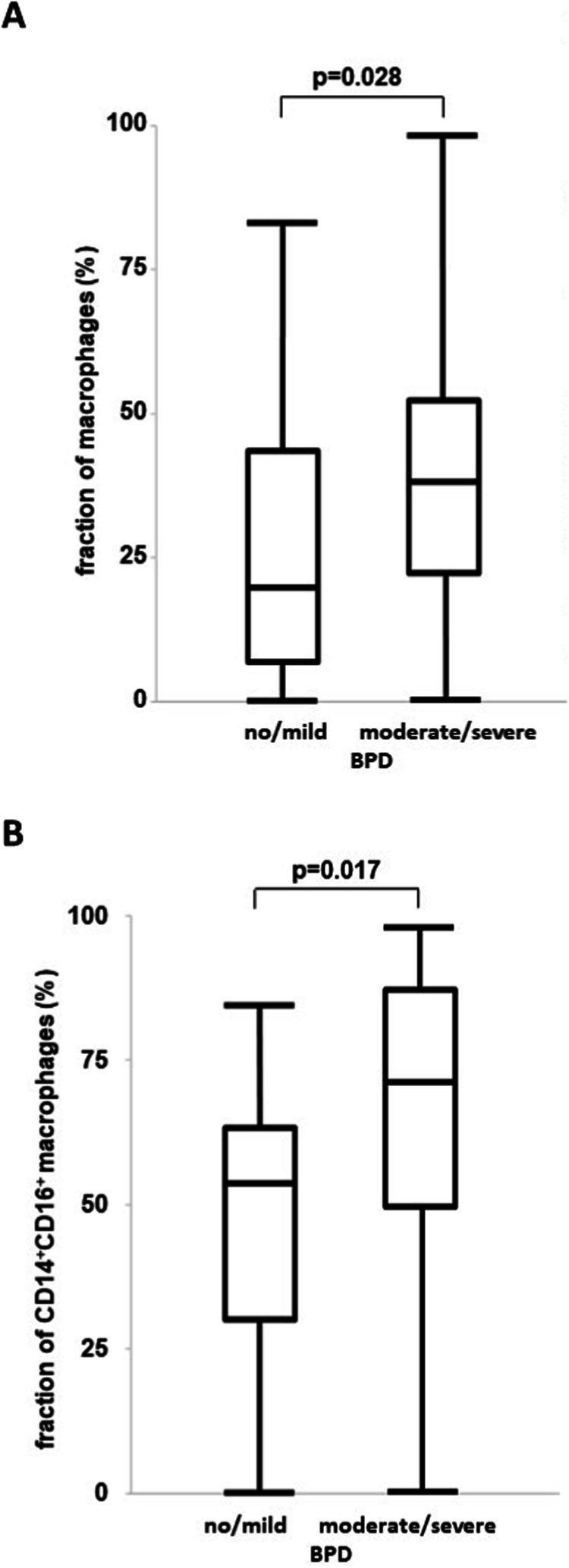
Higher fraction of CD14^+^CD16^+^ macrophages in tracheal aspirates of preterm infants associated with moderate/severe BPD. **A**, **B** Tracheal aspirates of *n* = 26 infants with no/mild BPD and *n* = 42 with moderate/severe BPD requiring prolonged mechanical ventilation until at least day 5 of life were analyzed for the fraction of macrophages (**A**) and CD14^+^CD16^+^ macrophage subtype (**B**). The fraction of macrophages (**A**) and the CD14^+^CD16^+^ macrophage subtype (**B**) were significantly higher in infants developing moderate/severe BPD. Data are presented as median, interquartils and range. Statistical analysis was performed using rank sum test. *p < 0.05.

We separated infants into those with a better (no and mild BPD) and worse (moderate and severe BPD) pulmonary outcome [29] and *n* = 42 developed moderate/severe BPD (Table 1). After 5 days of exposure to MV with HOX, flow cytometry analyses prevailed a predominance >90% of macrophages and neutrophils in the tracheal aspirates. Therefore, we focused our studies on the innate immune response. The innate immune cell distribution displayed a significantly higher fraction of total macrophages and a predominance of CD14^+^CD16^+^ macrophages in infants with moderate/severe BPD (Fig. 1A, B). These results formed the basis for our hypothesis that CCL2 mediated recruitment of macrophages to the oxygen exposed lung is a critical step in the pathogenesis of BPD.

**Table 1:** Baseline characteristics of infants still ventilated on day 5 of life.

	no/mild BPD (*n* = 26)	moderate/severe BPD (*n* = 42)
Gestational age	26 + 1 [24 + 1,28 + 3]	26 + 0 [22 + 5,28 + 6]
Birth weight [g]	775 [550, 1550]	690 [315,1215]
Female	10 (39%)	15 (36%)
Multiples	11 (42%)	19 (45%)
ANCS		
none/ < 24 h	6 (23%)	7 (17%)
≥24 h	20 (77%)	35 (83%)
Surfactant	22 (85%)	42 (100%)
Respiratory support [d]	53 [6,78]	76 [48,158]

Qualitative data is presented as n with proportion in brackets. Quantitative data is presented as median and range in square brackets. BPD bronchopulmonary dysplasia, ANCS antenatal corticosteroids; respiratory support comprises any form of invasive and non-invasive mechanical ventilation including highflow with a flow >2 l/min.

### Reduced lung injury in CCL2^−/−^ mice after exposure to 40% and 85% of oxygen

**Fig. 2:**
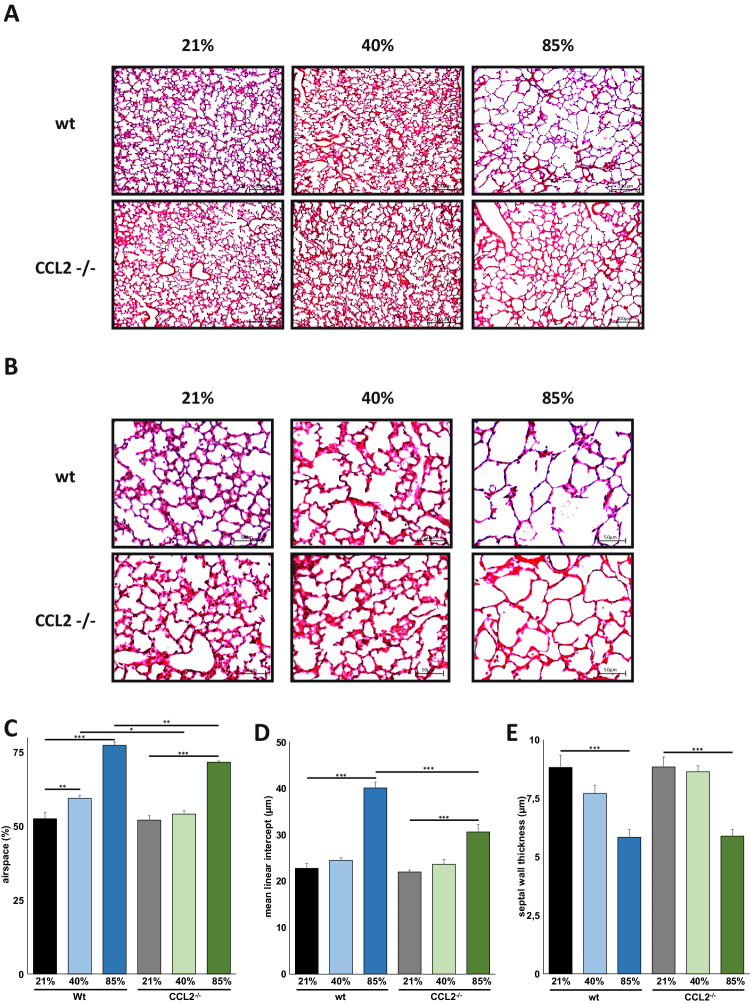
Hyperoxia-induced alveolar simplification is reduced in newborn CCL2^−/−^ mice. **A** Haematoxylin/eosin staining of representative lung tissue sections from newborn wildtype and CCL2^−/−^ mice exposed to 21%, 40% or 85% of oxygen for 7 days starting after birth. Hyperoxia induced alveolar simplification was visible at 40% and 85% of oxygen. Scale bar: 200 µm. **B** Enlarged image sections from haematoxylin/eosin stainings as in **A**. Scale bar: 50 µm. Corresponding lung morphometric analyses for airspace (**C**), mean linear intercept (**D**) and septal wall thickness (**E**) from **A**. Hyperoxia induced increase in airspace was significantly less pronounced in CCL2^−/−^ compared to wildtype mice for 40% and 85% of oxygen. For mean linear intercept, statistical significance was reached at 85% of oxygen. Data are presented as mean + SEM. Statistical analysis was performed by one way RM ANOVA. *p < 0.05, **p < 0.01, ***p < 0.001, ****p < 0.0001. *n* ≥ 6 mice/group.

To specify the consequences of hyperoxic lung injury, wildtype and CCL2^−/−^ mice were exposed to 40% or 85% of oxygen for the first 7 days of life modeling the clinical situation of preterm infants with less and more severe compromised gas exchange after birth. Survival rates until P8 did not differ between wildtype and CCL2^−/−^ animals exposed to 40% (88.9% versus 100%) and to 85% (100% versus 91.7%) of hyperoxia. As well, weight gain was not statistically significant different between the respective groups of wildtype and CCL2^−/−^ animals. For exposure to 85% of oxygen, median weight on P8 was 3.52 g (interquartile range 3.25–3.81, *n* = 17) grams in wildtype and 3.9 g (interquartile range 3.69–4.14, *n* = 13) grams in CCL2^−/−^ animals, with hyperoxia exposure to 40% in wildtype 3.995 g (interquartile range 3.547–4.46, *n* = 16) and in CCL2^−/−^ animals 3.62 g (interquartile range 3.5–3.8, *n* = 17) and in normoxic wildtype 3.29 g (interquartile range 3.02–3.48, *n* = 19) and CCL2^−/−^ animals 3.215 g (interquartile range 3.11–3.27, *n* = 16). Both in wildtype and transgenic animals, alveolar sacs were enlarged and mean linear intercept measurements were higher in oxygen exposed animals and the disruption of alveolar structures was higher in mice exposed to 85% HOX than in those to 40% HOX (Fig. 2A, B). Automated morphometric analyses prevailed that enlargement in airspaces at 85% HOX was significantly reduced in CCL2^−/−^ compared to wildtype animals. For the exposure to 40% HOX a comparable significant difference was present (Fig. 2C). In line, mean linear intercept was significantly reduced in CCL2^−/−^ compared to wildtype mice incubated with 85% HOX, while pathology in septal wall thickness did not differ between CCL2^−/−^ and wildtype animals (Fig. 2D, E).

### Reduced influx of innate immune cells and apoptosis induction in CCL2^−/−^ mice exposed to hyperoxia

**Fig. 3:**
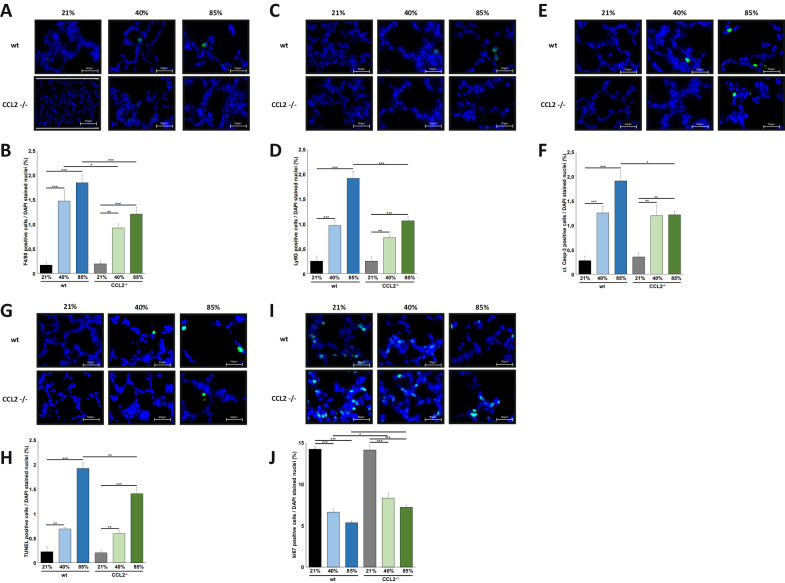
Hyperoxia-induced recruitment of innate immune cells and cell death induction are reduced in newborn CCL2^−/−^ mice. **A** Immunofluorescence staining for F4/80 positive macrophages in lung tissues from mice from Fig. 2A. Hyperoxia mediated influx of macrophages into the lungs of newborn mice was detectable at 40% and 85% of oxygen but was markedly prohibited in CCL2^−/−^ mice. Scale bar: 50 µm. **B** Quantification of F4/80 positive macrophages from **A**. Hyperoxia mediated influx of macrophages was significantly reduced in CCL2^−/−^ compared to wildtype animals at 40% and 85% of oxygen. **C** Immunofluorescence staining for Ly6G positive neutrophils in lung tissues from mice from Fig. 2A. Hyperoxia mediated influx of neutrophils into the lungs of newborn mice was demonstrated at 40% and 85% of oxygen but was markedly prohibited in CCL2^−/−^ mice at 85% of oxygen. Scale bar: 50 µm. **D** Quantification of Ly6G positive neutrophils from **C**. Hyperoxia mediated influx of neutrophils was significantly lower in CCL2^−/−^ compared to wildtype animals at 85% of oxygen. **E** Immunofluorescence staining for cleaved Caspase-3 (cl-Casp-3) positive cells in lung tissues from mice from Fig. 2A. Cleavage of Caspase-3 was present in the lungs of newborn mice exposed to 40% and 85% of oxygen. Scale bar: 50 µm. **F** Quantification of cl-Casp-3 positive cells from **E**. Hyperoxia induced cleavage of Caspase-3 was significantly reduced in CCL2^−/−^ compared to wildtype animals at 85% of oxygen. **G** Immunofluorescence staining for TUNEL positive cells in lung tissues from mice from Fig. 2A. Hyperoxia induced apoptosis induction in the lungs of newborn mice was apparent at 40% and 85% of oxygen. Scale bar: 50 µm. **H** Quantification of TUNEL positive cells from **G**. Hyperoxia mediated apoptosis induction was significantly increased in CCL2^−/−^ compared to wildtype animals at 85% of oxygen. **I** Immunofluorescence staining for Ki67 positive cells of lung tissues from mice from Fig. 2A. Hyperoxia induced reduction of Ki67 positive cells was present at 40% and 85% of oxygen. Scale bar: 50 µm. **J** Quantification of Ki67 positive cells from **I**. Hyperoxia induced reduction of Ki67 positive cells was significantly lower in CCL2^−/−^ compared to wildtype animals at 40% and 85% of oxygen. Data are presented as mean + SEM. Statistical analysis was performed by one way RM ANOVA. *p < 0.05, **p < 0.01, ***p < 0.001, ****p < 0.0001. *n* ≥ 6 mice/group.

We first documented the reduced influx of macrophages into the lung tissue in CCL2^−/−^ mice exposed to hyperoxia by immunofluorescence staining with F4/80. Compared to wildtype mice, CCL2^−/−^ animals had significantly less macrophages in their lungs both after exposure to 40% and 85% HOX for 7 days (Fig. 3A, B). This was accompanied by a significantly reduced influx of neutrophils in CCL2^−/−^ mice (Fig. 3C, D). The reduced inflammatory response in the lungs went along with attenuated apoptosis induction. Both the extent of Caspase-3 cleavage and the number of TUNEL positive cells were significantly lower in CCL2^−/−^ animals exposed to 85% HOX compared to their wildtype counterparts (Fig. 3E–H). Vice versa, Ki67 staining reflecting the proliferative activity prevailed significantly less attenuated in CCL2^−/−^ mice compared to wildtype mice when exposed to 40% or 85% HOX (Fig. 3I, J).

Taken together, the loss of CCL2 reduced the number of macrophages in the lungs following oxygen exposure which in turn resulted in lower neutrophil attraction and cell death induction but better-preserved lung proliferative capacity. Therefore, we next aimed to determine which lung cell populations benefited most from the prohibited macrophage recruitment to the HOX exposed lungs in CCL2^−/−^ mice.

### Better preserved lung cell populations in CCL2^−/−^ mice after exposure to 40% and 85% of oxygen

**Fig. 4:**
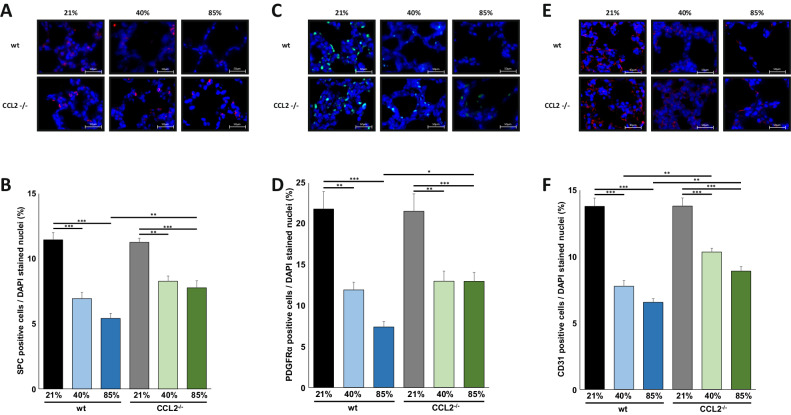
Better preserved lung cell populations in newborn CCL2^−/−^ mice exposed to hyperoxia. **A** Immunofluorescence staining for surfactant protein C (SPC) positive cells in lung tissues from mice from Fig. 2A. Hyperoxia induced rarefication of SPC cells was detectable at 40% and 85% of oxygen. Scale bar: 50 µm. **B** Quantification of SPC positive cells from (**A**). Hyperoxia induced rarefication of SPC positive cells was significantly less pronounced in CCL2^−/−^ compared to wildtype mice for 85% of oxygen. **C** Immunofluorescence staining for platelet derived growth factor receptor α (PDGFRα) positive cells in lung tissues from mice from Fig. 2A. Hyperoxia induced rarefication of PDGFRα cells was visible at 40% and 85% of oxygen. Scale bar: 50 µm. **D** Quantification of PDGFRα positive cells from (**C**). Hyperoxia induced rarefication of PDGFRα positive cells was significantly more pronounced in CCL2^−/−^ compared to wildtype mice for 85% of oxygen. **E** Immunofluorescence staining for vascular endothelial CD31 positive cells in lung tissues from mice from Fig. 2A. Hyperoxia induced rarefication of CD31 cells was apparent at 40% and 85% of oxygen. Scale bar: 50 µm. **F** Quantification of CD31 positive cells from (**E**). Hyperoxia induced rarefication of CD31 positive cells was significantly higher in CCL2^−/−^ compared to wildtype mice for 40% and 85% of oxygen. Data are presented as mean + SEM. Statistical analysis was performed using one way RM ANOVA. *p < 0.05, **p < 0.01, ***p < 0.001, ****p < 0.0001. *n* ≥ 6 mice/group.

As previously, we separately investigated the effects of exposure to 40% and 85% HOX for 7 days on the number of surfactant protein C (SPC) positive type II cells, platelet-derived growth factor receptor α (PDGFRα) positive mesenchymal stem cells (MSC) and CD31 positive vascular endothelial cells [16]. Immunofluorescence staining revealed that both 40% and 85% HOX significantly reduced the number of type II cells, MSC and vascular endothelial cells both in wildtype and CCL2^−/−^ mice and as expected, the effect size was larger at 85% HOX. The rarefication of all three lung cell populations was significantly more pronounced in wildtype than in CCL2^−/−^ mice exposed to 85% HOX. At 40% HOX, identical tendencies were present but statistical significance was solely reached for CD31 positive cells (Fig. 4A–F). Semiquantitative analyses displayed that the effect size was comparable for the type II cell population, MSC and the lung small vessels documenting the benefits of loss of CCL2 for the alveolar, mesenchymal and vascular compartment (Fig. 4A–F).

Taken together, the data obtained from biosamples of preterm infants and newborn mice allow the conclusion that lung macrophage recruitment contributes to hyperoxic lung injury and constitutes a key feature in the pathogenesis of BPD. Targeting CCL2 prevailed as a promising therapeutic approach to reduce the extent of hyperoxic injury which we finally tested in the newborn wildtype mouse model.

### Treatment with CCL2 blocking antibody alleviates the lung pathology in wildtype mice exposed to 85% of oxygen

**Fig. 5:**
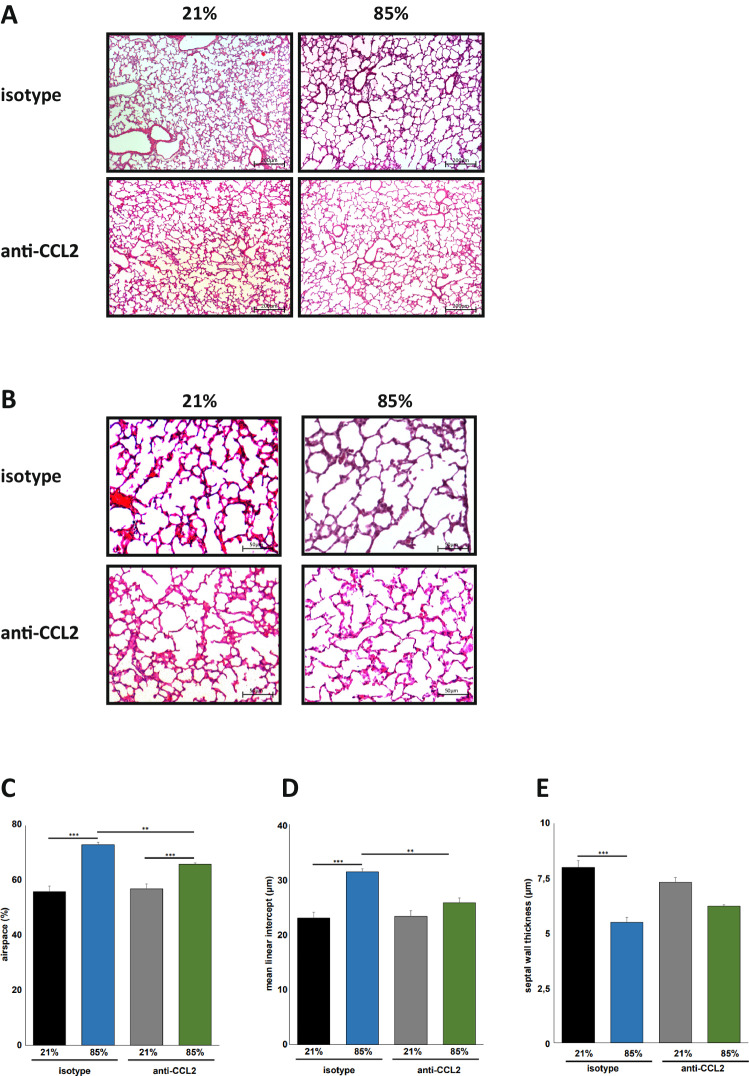
Pretreatment with CCL2 blocking antibodies attenuates hyperoxia induced lung injury in newborn wildtype mice. **A** Haematoxylin/eosin staining of representative lung tissue sections from newborn wildtype mice pretreated with isotype control or CCL2 blocking antibodies (10 µg/g body weight) within the first 12 h after birth before exposure to 21% or 85% of oxygen for 7 days. Treatment of mice with isotype control or CCL2 blocking antibodies was repeated on P3 and P5 of life. Hyperoxia induced alveolar simplification was visibly reduced in animals treated with CCL2 blocking antibodies. Scale bar: 200 µm. **B** Enlarged image sections from haematoxylin/eosin stainings as in (**A**). Scale bar: 50 µm. Corresponding lung morphometric analyses for airspace (**C**), mean linear intercept (**D**) and septal wall thickness (**E**) from (**A**). Hyperoxia induced increase in airspace and mean linear intercept was reduced in mice treated with CCL2 blocking antibodies. Data are presented as mean + SEM. Statistical analysis was performed using one way RM ANOVA. *p < 0.05, **p < 0.01, ***p < 0.001, ****p < 0.0001. *n* ≥ 4 mice/group.

**Fig. 6:**
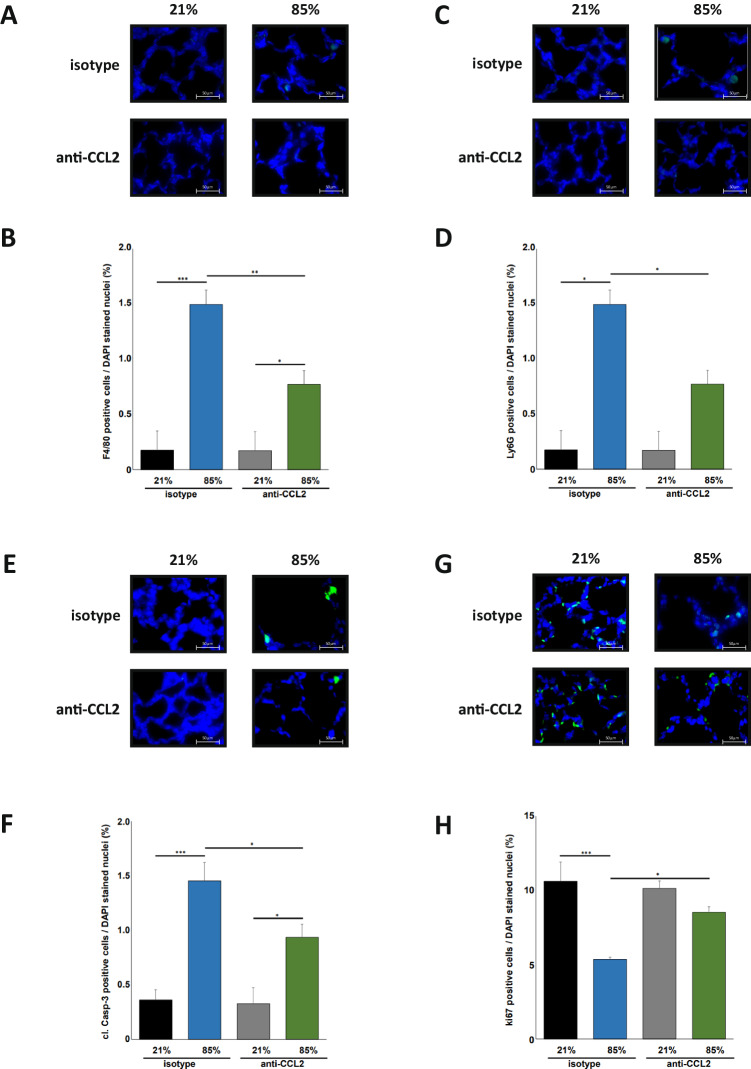
Reduced innate immune cell recruitment and cell death induction in newborn hyperoxia exposed wildtype mice treated with CCL2 blocking antibodies. **A** Immunofluorescence staining for F4/80 positive macrophages in lung tissues from mice from Fig. 5A. Hyperoxia mediated influx of macrophages into the lungs of newborn mice was markedly prohibited in wildtype mice treated with CCL2 blocking antibodies. Scale bar: 50 µm. **B** Quantification of F4/80 positive macrophages from (**A**). Hyperoxia mediated influx of macrophages was significantly reduced in wildtype mice treated with CCL2 blocking antibodies compared to isotype control at 85% of oxygen. **C** Immunofluorescence staining for Ly6G positive neutrophils in lung tissues from mice from Fig. 5A. Hyperoxia mediated influx of neutrophils into the lungs of newborn mice exposed to 85% of oxygen was markedly prohibited in mice treated with CCL2 blocking antibodies. Scale bar: 50 µm. **D** Quantification of Ly6G positive neutrophils from (**C**). Hyperoxia mediated influx of neutrophils was significantly lower in wildtype animals treated with CCL2 blocking antibodies and exposure to 85% of oxygen. **E** Immunofluorescence staining for cleaved Caspase-3 (cl-Casp-3) positive cells in lung tissues from mice from Fig. 5A. Increase in cleavage of Caspase-3 was visibly reduced in the lungs of newborn mice exposed to 85% of oxygen treated with CCL2 blocking antibodies. Scale bar: 50 µm. **F** Quantification of cl-Casp-3 positive cells from (**E**). Hyperoxia induced cleavage of Caspase-3 was significantly reduced in wildtype mice treated with CCL2 blocking antibodies compared to isotype control application. **G** Immunofluorescence staining for Ki67 positive cells in lung tissues from mice from Fig. 5A. Reduction of Ki67 positive cells was present in newborn mice exposed to 85% of oxygen but Ki67 positive cells were visibly better preserved when treated with CCL2 blocking antibodies. Scale bar: 50 µm. **H** Quantification of Ki67 positive cells from Fig. 5I. Hyperoxia induced reduction of Ki67 positive cells was significantly lower in wildtype animals exposed to 85% of oxygen when treated with CCL2 blocking antibodies. Data are presented as mean + SEM. Statistical analysis was performed by one way RM ANOVA. *p < 0.05, **p < 0.01, ***p < 0.001, ****p < 0.0001. *n* ≥ 4 mice/group.

We intraperitoneally injected CCL2 blocking antibodies or isotype control antibodies within 12 h after birth and before start of 85% HOX exposure for 7 days. Antibody application was repeated 48 and 96 h after start of HOX. Isotype treated mice displayed equivalent structural simplifications with enlarged airspaces and increased mean linear intercept and no significant changes in septal wall thickness when exposed to 85% HOX as wildtype animals without any treatment (Figs. 1A–E and 5A–E). Treatment with the CCL2 blocking antibody significantly reduced both increases in airspace and mean linear intercept but did not alter the HOX induced reduction in septal wall thickness (Fig. 5A–E). Efficacy of the therapeutic approach to attenuate macrophage recruitment to the HOX exposed lungs was documented by immunofluorescence staining and the number of macrophages was significantly reduced compared to isotype treated mice (Fig. 6A, B). As in the CCL2^−/−^ situation, the concomitant influx of neutrophils was also significantly attenuated (Fig. 6C, D). Caspase-3 cleavage as markers of lung injury was significantly reduced while lung cell proliferation measured by ki76 staining was better preserved (Fig. 6E–H). Effect sizes of treated animals were comparable as seen in the CCL2^−/−^ situation.

The data underpin that targeting macrophage recruitment to the immature lung alleviates the pathologic changes induced by HOX.

## Discussion

The large number of clinical and experimental research works has convincingly linked HOX induced pulmonary inflammation and lung injury to the development of BPD and lifelong restrictions in lung function in preterm infants [1, 3, 5]. Despite all the advances in scientific knowledge on this topic, the therapeutic approaches to reduce the BPD burden remain scarce and of limited efficacy even more than 50 years after the original description of BPD. Results from experimental studies during the recent years delivered the pathomechanistic explanation for the lack of effectiveness as inflammatory and lung growth promoting pathways coincide. This is why targeting pro-inflammatory cytokines like IL6, TNFα and TGFβ does not reduce the lung injury [13, 14, 30]. Within our translational approach, we identified inflammatory lung macrophage recruitment in preterm infants as particularly attractive cellular target and provide preclinical evidence that targeting macrophage recruitment by blocking CCL2 function constitutes a promising approach that preserves lung development during HOX exposure. Our results alleviate concerns from previous studies that imbalance in macrophage subpopulations can aggravate HOX induced lung injury [25, 26]. Rather, a reasonable explanation for the efficacy of targeting components of the cellular inflammatory response is that this approach does not interfere with physiologic cytokine signaling but just diminishes the excess pro-inflammatory cytokines released from attracted immune cells.

### The vision of targeted therapies to prevent BPD

Targeted therapies to prevent BPD have attracted particular attention during the recent years based on their specific mode of action and hopefully thereby avoiding the harms of broader acting substances including corticosteroids that are highly potent suppressors of inflammation but at the expense of acute side effects and serious long-term consequences particularly for the psychomotor outcome [31, 32]. As excess inflammation reduces lung growth factor cytokine levels, the disbalance of the cytokine equilibrium is further shifted into the wrong direction. Several strategies were evaluated within the rodent hyperoxia model and particularly IGF1, FGF10 and PDGFAA proved efficacy to reduce the alterations in lung morphometry. But so far only the first candidate was tested within a clinical trial not intended for BPD as primary readout and the results from an ongoing phase II study are still awaited [33–36]. Within the panel of therapeutic approaches towards attenuating the inflammatory response in the immature lung, targeting the inflammasome prevailed so far best suited and proved efficient in several preclinical studies, but still awaits documentation of its safety and efficacy in the clinics. Our results add a new promising category of strategies intended to reduce the influx of inflammatory cells. We preferred blocking antibodies against the CCL2 ligand over targeting its specific receptor CCR2 as it is well-established that CCR2 is not exclusively expressed on macrophages but as well on MSC, endothelial cells and even in the central nervous system on microglia and neurons where it exerts organ-protective and anti-inflammatory effects [37–39]. Furthermore, we aimed to circumvent receptor redundancy and compensation by another CCL2 ligand binding receptor while chemokine redundancy is not present to the same extent. Lastly, CCL2 function is not restricted to the CCR2 receptor and is much more complex than to be limited to a chemoattractant and to the macrophage cell population. It acts on neutrophils, B- and T-cells, NK cells and basophils and particularly comprises modification of inflammatory cell phenotypes including adhesion capacity, cell polarization, cytokine secretion and survival. The observed effect size of halving macrophage recruitment into the lung tissue is comparable to that observed in other disease models and of high clinical relevance [40].

### Putting our results into the context of the published literature

Our data are in line with the published literature from preterm infants that described association between the presence of inflammatory cells, particularly macrophages and neutrophils, in tracheal aspirates and BPD and between the influx of inflammatory cells into the lung tissue and stunting of further lung development in animal models of HOX exposure. Thereby, specific transcriptomic profiling signatures arising during the first week of life seem to be of particular relevance to the development of BPD [41]. The observations of increased levels of CCL2 in tracheal aspirate samples during mechanical ventilation with HOX in infants developing BPD is another indication for the relevance of lung macrophage attraction in the pathogenesis of BPD [20–22]. Our new results are congruent with a previous publication that together document comparable systemic and local macrophage subtype dynamics associated with BPD which is in line with the observation that specific inflammatory changes both in the lung and in the peripheral blood are associated with BPD [3, 27, 42, 43]. Further separation of macrophages based on their diverse function into Ly6C^low^ and Ly6C^high^ macrophages was not feasible as Ly6C is expressed on both subpopulations [44]. Reduction of alveolar type II cells, MSC and vascular endothelial cells following the HOX exposure was accompanied by the increase in cell death induction arguing for a relevant contribution to the rarefication in the lungs. Our recently published detailed studies on the effects of HOX on MSC are in line with the previous literature on this topic and argue for the contribution of both inhibition of proliferation and direct cytotoxicity by HOX [16].

### Strengths and limitations of the study

The major strength of our study is that it expands the knowledge on targeting CCL2 to prevent BPD and simultaneously refutes the concerns arising from the diverse outcomes of targeting the CCL2-CCR2 axis in the hyperoxia rodent models as detailed in the introduction. Second, beneficial effects were present at low and high concentrations of HOX, reflecting the clinical situation of preterm infants with modest and severe restrictions in gas exchange. In general, lung morphometry and the critical lung cell populations of type II cells, MSC and vascular endothelial cells were better preserved but the lower magnitude of changes at 40% HOX prohibited to document statistical significance apart from airspace enlargement, lung cell proliferation and vascular endothelial cell numbers. Third, we used newborn mice at equal sex distribution and all animals exposed to 85% HOX prevailed lower macrophage influx, lung injury and better-preserved lung morphometry under the condition of CCL2 deficiency or when treated with CCL2 blocking antibodies confirming the applicability to both sexes. Fourth, targeting CCL2 proved benefits not only for the alveolar and mesenchymal compartment but equally for vascularization of the lung which is critical for treatment efficacy as infants with BPD are particularly affected by stunted alveolarization and at long-term risk when alveolar simplification is accompanied by impaired pulmonary vessel formation and pulmonary hypertension [45]. But we need to acknowledge as well limitations of our results: The newborn hyperoxia mouse model varies from the situation of care for preterm infants that the majority of them requires supplemental oxygen after birth for normal oxygen saturation in contrast to the newborn mice. But the lungs of these mice have a comparable developmental stage and display highly comparable inflammatory response to hyperoxia and changes in lung pathology and in lung cell populations as observed in preterm infants [13, 16, 46]. The results in our translational study approach are furthermore substantiated by the congruency of results in preterm infants and newborn mice. Second, we did not study the long-term outcome after CCL2 blocking therapy and thereby cannot exclude unwanted side effects on the outcomes relevant for the situation of premature birth. But our study approach was intended towards efficacy not to cover all safety aspects in such detail. This will be priority within our subsequent studies. Third, CCL2 blockade is known to prime cell response to infection and to reduce cell-killing properties which are underdeveloped in preterm infants and are thereby at increased risk for nosocomial infections due to their immature immune system. Nevertheless, it will prerogative in any clinical study to survey infection rates following CCL2 antibody therapy. Fourth, CCL2 blocking antibodies were tested for indications in other adult inflammatory conditions without documenting efficacy. This was amongst other explanations traced back to the diverse immunomodulatory functions of CCL2 [47, 48]. Conversely, several CCL2 blocking antibodies tested for other medical indications are shortly available to be tested in preterm infants. Here, the rudimentary immune system of the preterm infant might have an advantage as its functionality is mainly based on the innate immune cells of monocytes/macrophages and neutrophils. Efficiently targeting inflammation might be more easily accomplished here than in adults with their complex immune physiology. Fifth, our data do not allow to separate whether the reduced neutrophil attraction to the lung was co-mediated by blocking CCL2 or by the mitigated lung inflammation. But in any case, CCL2 deficiency and CCL2 blocking therapy proved beneficial to reduce the influx of both key inflammatory leukocyte populations.

### Summary and conclusions

Taken together, our data provide novel pathomechanistic insights into lung macrophage recruitment and their subtype dynamics and BPD. Our targeted approach against CCL2 proved efficient to reduce lung inflammation and injury and thereby stands in contrast to the previous studies with disappointing outcomes. Our data confirm that targeting the inflammatory response during hyperoxic lung injury remains a promising research priority to improve the pulmonary outcome but document the need to identify the appropriate targets as done here for CCL2 or recently for the inflammasome which both do not interfere with physiologic lung signaling. The results can serve as basis to focus future BPD research efforts onto inflammatory cell recruitment which we recently ascribed additional capacity for lung injury beyond the direct toxic effects of HOX [16]. The positive results put general research priority on CCL2 signaling and can be of value for other lung diseases of all ages and beyond where activation of the CCL2/CCR2 axis is a hallmark event of disease pathology as described for example in influenza virus infection, cystic fibrosis and pulmonary fibrosis [49–51].

## Materials and methods

For immunofluorescence staining, anti-SPC (1:500, proSP-C) was purchased from Merck Millipore (Burlington, MA, USA), anti-Ki67 (1:200, SP6) from Novus Biologicals (Littleton, CO, USA), anti-CD31 (1:100, D8V9E), anti-PDGFRα (1:500, D1E1E) and anti-cleaved-Casp-3 (1:200, Asp175) from Cell Signaling, anti-PDGFRα from Santa Cruz (1:50, C-20), anti-F4/80 (1:100, ab6640) and anti-Ly6G (1:50, ab2557) antibody from abcam (Cambridge, UK), all secondary antibodies from Thermo Fisher Scientific. Signal intensity of cleaved-Casp-3 staining was amplified with signal stain boost IHC detection reagent (Cell Signaling) to improve signal detection. Cell surface receptor staining was executed with CD14-APC-Alx750 (1:50; MHCD1427), CD16-Alx700 (1:50; MHCD1629), CD45-APC-Alx750 (1:50; MHCD4527) and CD45-Pe-Cy5.5 (1:50;PSI-98-782) from Thermo Fisher Scientific (Waltham, MA), CD15-FITC (1:50; 130-081-101) from Miltenyi (Bergisch Gladbach, Germany), CD16-Alx700 (1:50; MHCD1629), CD45-APC-Alx750 (1:50; MHCD4527) and CD45PB (1:50; PB986) from Dako (Santa Clara, CA). All isotype controls were purchased from Beckton Dickinson besides mouse IgM-FITC isotype control (Miltenyi). All antibodies used in this manuscript can be found in SciCrunch database. CCL2 blocking antibody (BE 0185) and polyclonal Armenian hamster IgG (BE0091) isotype control for in vivo treatment studies were obtained from BioXCell (Lebanon, NH).

### Hyperoxia exposure of newborn mice

#### Transgenic animals

CCL2 knockout mice (B6.129S4-Ccl2^tm1Rol^/J) and C57BL/6 J wild type control animals were obtained from Charles River (Sulzfeld, Germany) [52].

#### Animal procedures

Newborn mice were exposed within the first twelve hours of life to oxygen concentrations of 21%, 40% or 85% at equal gender for seven days as described before [16]. Animals were sacrificed after 7 days of hyperoxia by intraperitoneal injection of a lethal dose of pentobarbital. Lungs were intracardially perfused with PBS at a pressure of 20 cmH_2_0, harvested en bloc for further analyses and directly frozen at −80 °C. Alternatively, lungs were fixed with intratracheal perfusion with buffered 4% paraformaldehyde (PFA) solution after binding the trachea with a string at a pressure of 20 cmH_2_O followed by placement in 4% PFA for 24 h at 4 °C. Lungs were then progressively dehydrated (30%, 50%, 70%, 99.6% ethanol) for 3 h each and embedded using a Leica embedding machine (EG 1150 C). Nursing dams were rotated between normoxia and 85% of hyperoxia at 12 h intervals and no rotation was executed at 40% of oxygen exposure. All scientific procedures on living animals were approved by the regional council (RP Giessen Az: V 54 - 19 c 20 15 h 01 GI20/12 Nr. 64/2018) in accordance with the German animal welfare law and the European legislation for the protection of animals used for scientific purposes (2010/63/EU). Investigators were not blinded to the treatment of the animals.

#### Therapeutic approach with blocking antibodies

Newborn mice were injected intraperitoneally with CCL2 blocking antibody or isotype control at 10 µg per gram body weight and after dilution in PBS to a total volume of 10 µl per gram body weight within 6 h after birth after skin disinfection with octenidine using a 30 G needle. Hyperoxia exposure for 7 days was started 6 h later and executed as described above. Antibody injections were repeated on P3 and P5.

#### Lung histology and immunofluorescence

Paraffin-embedded lungs were kept at 4 °C and 2 or 5 μm slices were cut thereof for haematoxylin and eosin and immunofluorescence staining. Haematoxylin and eosin stainings were analysed for mean linear intercept, air space and mean septal wall thickness from scans from the right upper lobe using a Leica DM6000B microscope with an automated stage and Qwin V3 software (Leica, Wetzlar, Germany) as before [52–54]. Bronchi and vessels with a diameter >50 µm were excluded from the analysis.

Deparaffinization and antigen unmasking paraffin sections for immunofluorescence staining was executed by incubation of lung slides in boiling 0.93% citrate buffer solution with a pH of 6 for 15 min for staining with anti-KI-67, anti-CD31, anti-PDGFRα, anti-cleaved-Casp-3 and anti-Ly6G or by treatment with proteinase K for 10 min for staining with anti-F4/80. Slides were blocked with 3% bovine serum albumin (BSA) and 0.4% Triton X-100 [in Tris-buffered saline (TBS)] at room temperature (RT) for 1 h before incubation with the primary antibodies overnight at 4 °C. Thereafter, slides were washed three times in TBST for 5 min followed by incubation with secondary antibodies for 1 h at room temperature. They were then washed three times in TBST before being mounted with Prolong Gold Anti-fade Reagent with DAPI (Thermo Fisher Scientific). TUNEL staining (Promega, Madison, WI) was performed as recommended by the manufacturer. Acquisition of fluorescent images was done using a Leica DM5500 B fluorescence microscope connected to a DFC360 FX camera (Leica). Quantification of immunofluorescence stainings was executed as described before and the number of positive cells was set against the number of DAPI stained nuclei [16].

### Fraction of macrophages and activation status in tracheal aspirates of preterm infants

Tracheal aspirates were collected from clinical routine suctioning in a cohort of *n* = 103 preterm infants <29 weeks´ gestation who failed successful stabilization on non-invasive ventilation at birth. *n* = 11 infants were excluded for death before 36 weeks´ gestation where the BPD severity status could not be determined. Subsequent samples for analyses of the effects of mechanical ventilation with oxygen rich gas were available on day 5 from infants that required prolonged mechanical ventilation. Severity stage of BPD was determined according to the 2001 NICHD BPD consensus definition [55].

Cell pellets of tracheal aspirates were centrifuged within 1 h after collection at 1000 g for 5 min and the supernatant was discarded. The pellet was resuspended in 100 µl of PBS supplemented with 2% fetal calf serum, 500 µl 1 M HEPES buffer solution, 0.02% sodium acide, 1 g/l glucose and 1 mM ethylenediaminetetraacetic acid. To specify macrophage subpopulations and neutrophils by cell surface marker staining, cells were incubated with CD14, CD15, CD16 and CD45 antibodies for 30 min on ice in the dark. Dead cells were excluded by propidium iodid staining (1 µg/ml). Flow cytometry was executed on a LSR-II device (Becton Dickinson, Heidelberg, Germany) with Diva software (Becton Dickinson) for data acquisition and FlowJo software (Tree Star Inc., Ashland, VA, USA) for all data analyses. Autofluorescence compensation was executed using an unstained control sample and anti-mouse Igκ and negative control compensation beads (Becton Dickinson) stained with each CD surface antibody. Due to the low sample volumes, staining with each CD surface receptor was not feasible.

### Ethics approval and consent to participate

The human studies were approved by the ethics committee of the Ludwig-Maximilians-University Munich (no. 195-07) in accordance with the declaration of Helsinki and written informed parental consent was obtained in all cases. The study was registered at the German Clinical Trials Register (DRKS00004600).

### Statistical analysis

All data are given as mean and SEM or median, interquartiles and range. The required sample size of *n* = 6 for each experimental setting in newborn mice was calculated using GPower version 3.1. at a significance level of *p* = 0.05 with an estimated effect size of 0.85 and a power >0.8. Student’s *t*-test, rank sum test or one way RM ANOVA with Holm–Sidak correction were used to test for statistically significant differences. Statistical analyses were performed using Sigma Plot 12.3. (Systat Software, San Jose, CA). Differences were considered significant at a *p* < 0.05.

## Data Availability

The datasets generated and analyzed during the current study are available from the corresponding author upon reasonable request.
